# FAME3R: an efficient, practical and reliable open-source tool for predicting phase 1 and phase 2 sites of metabolism

**DOI:** 10.1186/s13321-026-01161-1

**Published:** 2026-02-14

**Authors:** Roxane Axel Jacob, Leo Gaskin, Thomas Seidel, Ya Chen, Angelica Mazzolari, Johannes Kirchmair

**Affiliations:** 1 Department of Pharmaceutical Sciences, Faculty of Life Sciences, University of Vienna, Josef-Holaubek-Platz 2, 1090 Vienna, Austria; 2 Christian Doppler Laboratory for Molecular Informatics in the Biosciences, Department of Pharmaceutical Sciences, University of Vienna, Josef-Holaubek-Platz 2, 1090 Vienna, Austria; 3 Vienna Doctoral School of Pharmaceutical, Nutritional and Sport Sciences, University of Vienna, Josef-Holaubek-Platz 2, 1090 Vienna, Austria; 4 Dipartimento di Scienze Farmaceutiche, Universita degli Studi di Milano, 20133 Milano, Italy

**Keywords:** Xenobiotic metabolism, Metabolism prediction, Sites of metabolism

## Abstract

Predicting likely sites of metabolism (SOMs), i.e., the atoms in a molecule where metabolic reactions are initiated, is an important component of the computational development pipeline for pharmaceuticals, agrochemicals, and cosmetics. Among SOM prediction tools, FAME3, introduced in 2019, is one of only a few non-commercial models capable of predicting both Phase 1 and Phase 2 SOMs for a wide range of xenobiotics. However, its original implementation posed challenges in maintainability, scalability, and interoperability, which hindered broader adoption. To overcome these limitations, we developed FAME3R, an enhanced version of FAME3 designed to improve computational efficiency and facilitate integration with contemporary cheminformatics workflows. FAME3R introduces several new features, including a novel reliability assessment method based on Shannon entropy and the option to select among various featurization strategies. The tool is available as an open-source Python package, offering both a Python API and a CLI for flexible usage. Additionally, trained FAME3R models can be accessed via a GUI and a REST API hosted on the NERDD web platform.

**Supplementary Information:**

The online version contains supplementary material available at 10.1186/s13321-026-01161-1.

## Introduction

Understanding the metabolic fate of bioactive small molecules is crucial in drug discovery and development, as metabolism directly impacts a compound’s efficacy and safety [1]. Computational methods for predicting metabolism complement experimental techniques by providing rapid, cost-effective insights into drug-enzyme interactions, metabolic hotspots, and biotransformations. These *in silico* approaches help prioritize candidate compounds and optimize their metabolic stability and safety profiles [2–8].

Among available computational models, Site of Metabolism (SOM) predictors are widely used to identify and rank the atoms or bonds within a molecule most susceptible to metabolic reactions [9–14]. However, few tools extend beyond cytochrome P450 (CYP)-mediated metabolism to cover a broader range of enzymatic transformations. FAME3 [11] is a notable exception, offering comprehensive coverage of both phase 1 and phase 2 metabolic enzymes. It combines rooted circular fingerprints, topological and physicochemical descriptors (the *FAME descriptors*), and the extremely randomized trees algorithm, trained on expert-curated data from the MetaQSAR database [15], which includes over 2000 molecules with experimentally validated SOMs.

**Fig. 1:**
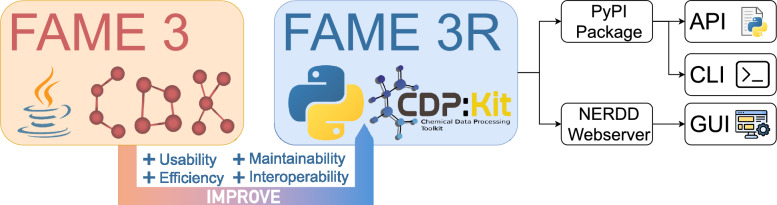
Overview of FAME3R functionalities. FAME3R is a re-design of the FAME3 model, designed to address challenges related to computational efficiency, usability, and interoperability. It is available as an open-source Python package, offering access to all features through an application programming interface and command-line tools. For predicting SOMs, pre-trained FAME3R models can be accessed via a graphical user interface on the NERDD web platform.

Despite its strengths, FAME3 has limitations in terms of maintainability, scalability, and integration with modern software environments, hindering its adoption in contemporary workflows. To address these issues, we developed FAME3R, a re-designed, open-source Python package that improves computational efficiency, usability, and interoperability. FAME3R provides access to all functionalities, including descriptor generation and model building, via an Application Programming Interface (API) and command-line tools. We provide pre-trained models for non-commercial research through the NERDD web platform [16, 17] or Zenodo for local deployment. Commercial users can access training datasets and models under a licensing agreement (Fig. 1).

## Implementation

The FAME3R Python library enables the development of FAME3-like models. This section provides an overview of its core components and innovations over FAME3.

### Descriptors

FAME3R implements descriptors combining rooted circular fingerprints, ten physicochemical descriptors, and four topological descriptors, collectively referred to as *FAME descriptors*. These descriptors capture structural, electronic, and spatial properties essential for predicting SOMs. While based on the descriptors defined in the original FAME3 implementation, their re-implementation in FAME3R introduces several enhancements that improve both functionality and adaptability.

A key development in FAME3R is the adoption of the Chemical Data Processing Toolkit (CDPKit) [18] to replace the Java-based Chemistry Development Kit (CDK) [19] used in FAME3. CDPKit provides a Python interface to the C++-based Chemical Data Processing Library (CDPL), ensuring computational efficiency and seamless integration with modern Python workflows. CDPKit-specific preprocessing tasks, such as calculating implicit hydrogen counts, hybridization states, aromaticity flags, and electronic properties required for descriptor generation, are all handled internally by the implementation.

FAME3R’s modular design allows the customization of descriptor generation, such as adjusting fingerprint radius or selecting specific descriptor subsets, thereby enhancing adaptability to diverse research needs.

#### Circular fingerprints

Rooted circular fingerprints encode the local chemical environment around each heavy atom in a molecule. These fingerprints are generated within a user-specified bond radius and are equivalent to the *FAME fingerprints* introduced in the FAME3 study [11]. Each atom is assigned one of 26 SYBYL atom types [20], which represent common chemical elements in organic molecules across various hybridization states (see Section S.1 for a list of SYBYL atom types used in this work).

The fingerprints are constructed by assigning a 32-bit vector to every combination of a SYBYL atom type and bond distance from a given atom. For each distance, the first *n* bits in the vector are set to 1 if the atom has *n* neighbors of a specific atom type at that distance (see Section S.2 for an illustrated example).

In addition to the *FAME fingerprints*, FAME3R supports the generation of conventional rooted circular count fingerprints. These count fingerprints retain the same informational content as *FAME fingerprints* but significantly reduce the sparsity of the feature vector. As shown in Section S.3, replacing *FAME fingerprints* with count fingerprints maintains the predictive performance of the SOM model while enhancing computational efficiency.

#### Physicochemical descriptors

FAME3R includes ten atomic physicochemical descriptors, originally implemented in FAME3 using CDK and re-implemented in FAME3R with CDPKit, to capture key chemical and electronic properties. Notably, the StabilizationCharge descriptor from CDK was replaced with the InductiveEffect descriptor for an improved representation of electronic effects. Table 1 summarizes these descriptors.

**Table 1:** FAME3R’s physicochemical descriptors and their corresponding function in CDPKit

Descriptor	CDPKit function
Atom degree	MolProp.getHeavyAtomCount
Formal valence	MolProp.calcExplicitValence
Hybrid polarizability	MolProp.getHybridPolarizability
VSEPR coordination geometry	MolProp.getVSEPRCoordinationGeometry
Effective polarizability	MolProp.calcEffectivePolarizability
Inductive effect	MolProp.calcInductiveEffect
PEOE sigma charge	MolProp.getPEOESigmaCharge
PEOE sigma electronegativity	MolProp.getPEOESigmaElectronegativity
Pi electronegativity	MolProp.calcPiElectronegativity
Partial charge (MMFF94 force field)	ForceField.getMMFF94Charge

#### Topological descriptors

FAME3R includes four topological descriptors capturing molecular topology and the relative position of atoms within a molecule. These descriptors include (1) the maximum bond distance between any two atoms in the molecule (longestMaxTopDistinMolecule), (2) the maximum bond distance from the described atom to any other atom in the molecule (highestMaxTopDistinMatrixRow), (3) the difference between these two bond distances, and (4) the ratio of these two bond distances.

### Applicability domain and predictive uncertainty quantification

#### FAME score

To estimate prediction reliability, the original FAME3 study [11] proposed a similarity-based applicability domain score known as the *FAME score*, which is defined as:

1 $$\begin{aligned} \text {FAMEScore}({{\textbf {x}}}) = \frac{1}{k} \sum _{i=1}^{k}{\text {Tanimoto}({{\textbf {x,x}}}_i)} \end{aligned}$$

where ${{\textbf {x}}}$ is the rooted circular fingerprint of the atom, and ${{\textbf {x}}}_i$ is the fingerprint of the *i*-th nearest neighbor in the training set. By default, we set $k=3$, matching the value used in the original FAME3 study. The Tanimoto similarity between two fingerprints ${{\textbf {a}}}$ and ${{\textbf {b}}}$ is computed as:

2 $$\begin{aligned} \text {Tanimoto}({{\textbf {a,b}}}) = \frac{{{\textbf {a}}} \cdot {{\textbf {b}}}}{||{{\textbf {a}}}||^2 + ||{{\textbf {b}}}||^2 - {{\textbf {a}}} \cdot {{\textbf {b}}}} \end{aligned}$$

where ${{\textbf {a}}} \cdot {{\textbf {b}}}$ is the dot product (i.e., the intersection of bits), and $||{{\textbf {a}}}||^2$ and $||{{\textbf {b}}}||^2$ are the squared norms (i.e., the total number of active bits in each vector). The denominator computes the union of bits between the two vectors.

*FAME scores* range from 0 to 1, where higher values indicate that an atom’s chemical environment is well-represented in the training data, and, consequently, familiar to the model. Despite its simplicity, this feature offers valuable insights into the model’s applicability domain, enabling users to evaluate the reliability of its predictions.

#### Shannon entropy

The *FAME score* is a descriptor-space proximity metric and thus does not take into account the distribution of labels among the nearest neighbors. To overcome this limitation, we introduce an additional metric to assess the reliability of predictions: the Shannon entropy. This measure is defined as:

3 $$\begin{aligned} {\mathbb {H}} = -(p \cdot \textrm{log}_{2}(p) + (1 - p) \cdot \textrm{log}_{2}(1 - p)) \end{aligned}$$

where *p* represents the SOM probability output by the classifier. Shannon entropy values range from 0 to 1, with lower values indicating more reliable predictions. Although ${\mathbb {H}}$ does not aggregate over an explicit nearest neighbor set, it inherits the model’s locality: the model typically places greater weight on training samples that are more similar to the query, so ${\mathbb {H}}$ implicitly reflects local label homogeneity (low ${\mathbb {H}}$) or heterogeneity (high ${\mathbb {H}}$). Because ${\mathbb {H}}$ is a symmetric, monotonic transform of the distance of *p* from 0.5, it provides a concise, thresholdable indicator of uncertainty that is invariant to which class is favored. A detailed comparison between the *FAME score* and the Shannon entropy, including their mutual correlation and their correlation with predictive performance, is provided in the Supporting Information, Section S.4.

### Classification algorithm

FAME3R replaces the extremely randomized trees algorithm used in FAME3 with the random forest algorithm from the scikit-learn library. To convert predicted probabilities into binary predictions, a binary decision threshold is applied. While FAME3 used a threshold of 0.4, FAME3R adopts a threshold of 0.3 by default, which was found to provide more balanced results for models derived from the updated MetaQSAR data set. This adjustment reflects a deliberate effort to optimize the trade-off between precision and recall. More information can be found in Section S.5 of the Supporting Information.

## Usage

FAME3R can be operated via a Python API and command-line tools. Additionally, we provide a Graphical User Interface (GUI) through the NERDD web platform [16, 17] for researchers who prefer a non-programmatic approach to predicting SOMs with FAME3R. A description of each interface can be found in the Supporting Information, Section S.6. Detailed usage information can be found in the package’s online documentation [21].

## Performance

The predictive performance of FAME3R was evaluated using the Receiver Operating Characteristic Curve (ROC-AUC), Area Under the Precision Recall Curve (PR-AUC), F1-score, Matthews Correlation Coefficient (MCC), precision, recall, and Top-2 Correctness Rate (TOP-2). The results are summarized in Table 2. Our analysis considered four versions of FAME3 and FAME3R, each trained on a distinct subset of reactions: the entire training set, CYP-mediated reactions, Phase 1-associated reactions (reduction-oxidation and hydrolysis reactions), and Phase 2-associated reactions (primarily conjugation reactions). Both models were trained on the same rooted circular *FAME fingerprints*, ten physicochemical descriptors, and four topological descriptors. All predictions generated using FAME3 and FAME3R are fully deterministic. Detailed information on the data processing and splitting pipelines is available in the Supporting Information, Section S.7.

**Table 2:** Predictive Performance of FAME3R vs. FAME3 for Different Reaction Subsets

Data split	All reactions		CYP		Phase 1		Phase 2	
model	FAME3$^1$	FAME3R	FAME3$^1$	FAME3R	FAME3$^1$	FAME3R	FAME3$^1$	FAME3R
ROC-AUC	0.90	0.89	0.92	0.90	0.88	0.88	0.97	0.97
PR-AUC	n.a.	0.55	n.a.	0.64	n.a.	0.55	n.a.	0.77
F1 score	n.a.	0.54	n.a.	0.60	n.a.	0.53	n.a.	0.72
MCC	0.50	0.49	0.57	0.54	0.53	0.47	0.71	0.70
Precision	n.a.	0.49	n.a.	0.57	n.a.	0.46	n.a.	0.65
Recall	n.a.	0.61	n.a.	0.64	n.a.	0.63	n.a.	0.79
TOP-2	0.82	0.84	0.90	0.84	0.83	0.77	0.92	0.88

$^1$ Metrics sourced from the original FAME3 publication [11]

FAME3R models achieve predictive performances comparable to that of the original FAME3 models across all reaction subsets. The slight decrease in MCC and TOP-2 observed for CYP-mediated and Phase 1 reactions in FAME3R is likely attributable to differences in data preprocessing, particularly the stricter handling of stereoisomers in this study, which results in a more challenging test set (see Supporting Information, Section S.7). Despite this, the performance of FAME3R is comparable with that of FAME3, confirming its reliability.

In addition to achieving predictive performance comparable to FAME3, FAME3R offers substantial improvements in computational efficiency. For FAME3, the average runtime per atom is 78 ms without the *FAME score* and 823 ms with the *FAME score*. In contrast, FAME3R requires only 1.5 ms and 8.4 ms per atom under the same conditions, respectively. This corresponds to a 50-fold speedup without the *FAME score* and a remarkable 100-fold speedup when the *FAME score* is included. The computation of the Shannon entropy is highly efficient, resulting in negligible computational overhead. Runtimes were averaged per atom over the Phase 1 + Phase 2 test set, comprising 8860 heavy atoms across 393 molecules, and were measured on a single core of an AMD Ryzen 9 7950X processor. The FAME3 CLI supports multi-core execution for SOM prediction and can therefore benefit from parallelization proportional to available hardware. This was intentionally not re-implemented in the FAME3R CLI, as the single-core performance is already sufficient for typical use cases. Users wanting to apply FAME3R to truly large datasets are encouraged to make use of the provided Python library in order to parallelize FAME3R operations in a way that is optimal for their own hardware.

## Conclusions

In conclusion, FAME3R retains the state-of-the-art predictive accuracy of FAME3 while addressing its limitations in terms of scalability, efficiency, and usability. With its extended functionalities, including support for diverse fingerprints and reliability assessment, FAME3R is a versatile and practical tool for SOM prediction. Its release as an open-source Python package, coupled with seamless access via the NERDD web platform, makes it a valuable resource for advancing research in xenobiotic metabolism.

## Supplementary information

Supporting information. It includes the full list of SYBYL atom types used to compute the descriptors (Section S.1), an example of the construction of FAME fingerprints (Section S.2), a comparison of the performance of FAME3R models trained on FAME fingerprints vs. count fingerprints (Section S.3), a comparison of the FAME score and Shannon entropy as measures of prediction reliability (Section S.4), an investigation of the precision-recall trade-off (Section S.5), information on the use of FAME3R’s API and CLI (Section S.6), and detailed information on the data used to evaluate the FAME3 and FAME3R models (Section S.7).

## Data Availability

Trained FAME3R models are accessible through both a GUI and a REST API hosted on NERDD (https://nerdd.univie.ac.at/fame3r). The source code is open-source and available in the molinfo-vienna/FAME3R GitHub repository (https://github.com/molinfo-vienna/FAME3R). The software is platform-independent, written in Python, and distributed under the MIT license. Pre-trained FAME3R models, developed using the MetaQSAR database, are available for download from Zenodo (https://doi.org/10.5281/zenodo.17294556). These models are freely available for non-commercial research purposes. For-profit institutions must obtain a commercial license from the Università degli Studi di Milano to access the MetaQSAR-derived models.
