# Supplementary material for: FAME3R: an efficient, practical and reliable open-source tool for predicting phase 1 and phase 2 sites of metabolism
Source: J Cheminform. 2026 Feb 14;18:37. doi: 10.1186/s13321-026-01161-1 (PMC13011438; doi:10.1186/s13321-026-01161-1)
Supplement: Supplementary file 1 — Supporting information. It includes the full list of SYBYL atom types used to compute the descriptors (Section S.1), an example of the construction of FAME fingerprints (Section S.2), a comparison of the performance of FAME3R models trained on FAME fingerprints vs. count fingerprints (Section S.3), a comparison of the FAME score and Shannon entropy as measures of prediction reliability (Section S.4), an investigation of the precision-recall trade-off (Section S.5), information on the use of FAME3R’s API and CLI (Section S.6), and detailed information on the data used to evaluate the FAME3 and FAME3R models (Section S.7). [file 13321_2026_1161_MOESM1_ESM.pdf]

# – Brief Report –

FAME3R: An Efficient, Practical and Reliable Open-Source Tool for Predicting Phase 1 and Phase 2 Sites of Metabolism

Roxane Axel Jacob,[†,‡,§] Leo Gaskin,[†] Thomas Seidel,[†,‡] Ya Chen,[†] Angelica Mazzolari,[¶] and Johannes Kirchmair[*,†,‡]

†*Department of Pharmaceutical Sciences, Division of Pharmaceutical Chemistry, Faculty of Life Sciences, University of Vienna, Josef-Holaubek-Platz 2, 1090 Vienna, Austria*

‡*Christian Doppler Laboratory for Molecular Informatics in the Biosciences, Department of Pharmaceutical Sciences, University of Vienna, Josef-Holaubek-Platz 2, 1090 Vienna, Austria*

¶*Dipartimento di Scienze Farmaceutiche, Universita degli Studi di Milano, I-20133 Milano, Italy*

§*Vienna Doctoral School of Pharmaceutical, Nutritional and Sport Sciences, University of Vienna, Josef-Holaubek-Platz 2, 1090 Vienna, Austria*

E-mail: johannes.kirchmair@univie.ac.at

Table of Content

Sections

S.1 SYBYL Atom Types 3

S.2 FAME fingerprints 3

S.3 FAME Fingerprints vs. Count Fingerprints 4

S.4 FAME Score vs. Shannon Entropy 4

S.5 Binary Decision Threshold 5

S.6 Usage 6

S.7 Data 7

Tables

S1 SYBYL Atom Types and their CDPKit Designations 13

S2 Comparison of the Predictive Performance of FAME3R with FAME fingerprints vs. count fingerprints 13

S3 Comparison of the Composition of the Data Used in this Work vs. in the FAME3 Validation Study 14

S4 MetaQSAR Classification of Metabolic Reactions 15

S5 Composition of the Full, CYP-, Phase 1-, and Phase 2-Only Data Sets 16

Figures

S1 Illustration of the FAME fingerprint using omeprazole and a maximum radius of 3 as an example 17

S2 Comparison of FAME scores and Shannon entropy for prediction reliability 18

S3 Precision, recall and MCC vs. binary decision threshold 19

S4 Workflow diagram of the FAME3R command-line interface tools 20

S.1 SYBYL Atom Types

The 26 SYBYL atom types used in this work are listed in Table S1, along with their chemical descriptions and corresponding Chemical Data Processing Toolkit (CDPKit)[1] identifiers. These atom types provide a standardized classification of common elements and hybridization states encountered in organic molecules.

S.2 FAME Fingerprints

FAME fingerprints are circular fingerprints that encode the local chemical environment around each heavy atom in a molecule within a user-specified bond radius. These fingerprints, identical to those introduced in the FAME3 study,[2] assign one of 26 SYBYL atom types[3] to each atom, representing common elements in organic molecules across various hybridization states (see Table S1 for details).

Each fingerprint is constructed by assigning a 32-bit vector to every combination of a SYBYL atom type and bond distance from a given atom. For each distance, the first n bits in the vector are set to 1 if the atom has n neighbors of a specific atom type at that distance. The 32-bit strings for all radii are then concatenated to form the final fingerprint.

To illustrate, we use omeprazole as an example (Figure S1) with a maximum bond radius of 3 (default is 5 in FAME3R). The fingerprint is generated for the aromatic carbon atom (C.ar) highlighted in brown. At radius zero, the first bit of the C.ar vector is activated. At radius one, the atom has one sp^3-hybridized carbon (C.3) and two aromatic carbons, activating the first bit of the C.3 vector and the first two bits of the C.ar vector. This process is repeated for all radii up to the maximum, with no bits activated for absent atom types. For a radius of 3, the final fingerprint contains 4 radii (0 to 3) $\times$ 26 atom types $\times$ 32 bits = 3328 values per atom.

S.3 FAME Fingerprints vs. Count Fingerprints

In this section, we compare the predictive performance of two versions of the FAME3R model: one trained on data featurized using *FAME fingerprints* and the other trained on data featurized using conventional count fingerprints. Both models also incorporate physicochemical and topological fingerprints. The training data for both models includes Phase 1 and Phase 2 metabolic reactions. The results, presented in Table S2, indicate that the two fingerprinting methods yield nearly equivalent performance, with a slight advantage observed for the count fingerprints.

S.4 FAME Score vs. Shannon Entropy

In this section, we compare the *FAME scores* with the Shannon entropy values for each atom in the test set of the Phase 1 and Phase 2 model trained on *FAME descriptors* (fingerprints, physicochemical, and topological descriptors). Panels A and B in Figure S2 illustrate the distributions of *FAME scores* and Shannon entropies, respectively. Both measures are theoretically bounded between [0, 1]. However, due to the unlikeliness of low *FAME score* values, the observed minimum *FAME score* in this data set is 0.39. Higher *FAME scores* indicate more reliable predictions, whereas lower Shannon entropies correspond to greater reliability. To facilitate comparison, we inverted the x-axis of Panel B (the same goes for panel E, which will be explained in the following).

Panel C presents a scatter plot of Shannon entropy versus *FAME score*, revealing two key observations: (1) *FAME scores* tend to cluster in discrete intervals, unlike Shannon entropy, and (2) there is no strong correlation between the two measures. The Pearson correlation coefficient (quantifying linear correlation) is -0.33, while the Spearman correlation coefficient (quantifying rank-order correlation) is -0.44.

Panels D and E examine the Brier score for increasingly reliable prediction subsets, ranked by *FAME score* and Shannon entropy, respectively. Specifically, predictions are sorted by increasing unreliability, and the Brier score is computed for progressively larger subsets of the test set. The first subset includes only the most reliable predictions, while the final subset includes all predictions. The Brier score is a strictly proper scoring rule that measures the accuracy of probabilistic predictions. In our case, it is equivalent to the mean squared error as applied to the predicted Site of Metabolism (SOM) probabilities. A negative linear trend is expected, where stronger slopes indicate better alignment between the reliability measure and predictive performance. From Panels D and E, it is evident that Shannon entropy is a more effective measure of prediction reliability than the *FAME score*. This result is unsurprising, as Shannon entropy accounts for the distribution of labels among training set instances similar to the test instance, a factor not considered by the *FAME score*.

S.5 Binary Decision Threshold

The binary decision threshold is the value above which a probabilistic prediction is classified as a positive binary prediction. This threshold can be adjusted to influence key performance metrics such as precision and recall. Precision measures the proportion of correct positive predictions out of all positive predictions made by the model, while recall measures the proportion of correct positive predictions out of all actual positive instances in the data set. These two metrics are inherently in trade-off: increasing the threshold typically improves precision but reduces recall, and vice versa. In many cases, users aim to strike a balance between precision and recall. This can be quantified using metrics that are optimized when precision and recall are balanced out, such as the Matthews Correlation Coefficient (MCC). Figure S3 illustrates how precision, recall, and MCC vary with the binary decision threshold for predictions generated by the Phase 1 and Phase 2 FAME3R model on the Phase 1 and Phase 2 test set.

S.6 Usage

S.6.1 Application Programming Interface

The FAME3R Python Application Programming Interface (API) provides a scikit-learn[4]-compatible interface for computing the *FAME descriptors* and the *FAME scores* of small organic molecules. The API is designed to be modular, flexible, and easily integrable into existing cheminformatics workflows. It consists of two main components: the `FAME3RVectorizer` for feature generation and the `FAME3RScoreEstimator` for applicability scoring. The following code snippet demonstrates a typical usage workflow:

```
import numpy as np
from sklearn.ensemble import RandomForestClassifier
from fame3r import FAME3RVectorizer, FAME3RScoreEstimator

# Initialize and fit the vectorizer
vectorizer = FAME3RVectorizer(
    radius=5,
    input="smiles",
    output=[
        "fingerprint",
        "physicochemical",
        "topological"
    ]
)
vectorizer.fit()

# Transform input fragments to feature vectors
```

```python
    smiles_fragments = [["CC[C:1]"], ["CC[N:1]"], ["CC[O:1]"]]
    features = vectorizer.transform(smiles_fragments)

    # Initialize and fit a SOM prediction model
    # FAME3R uses scikit-learn's RandomForestClassifier,
    # but any machine learning model could be used instead
    som_predictor = RandomForestClassifier()
    som_predictor.fit(features, labels=[0, 0, 1])

    # Initialize and fit the FAME score estimator
    fame_score_estimator = FAME3RScoreEstimator(n_neighbors=3)
    fame_score_estimator.fit(features)

    # Predict SOMs and FAME scores for new molecules
    new_fragment = [["[C:1]CC"]]
    new_features = vectorizer.transform(new_fragment)
    predicted_soms = som_predictor.predict(new_features)
    fame_scores = fame_score_estimator.predict(new_features)
```

The `FAME3RVectorizer` adheres to scikit-learn's fit/transform API convention and is used for converting atomic environments into feature vectors, also known as *FAME descriptors*, which are detailed in the *Descriptors* subsection. The `FAME3RVectorizer` can be initialized with the following parameters:

- `radius`: Specifies the bond radius used for generating circular fingerprints (default: 5).
- `input`: Defines the format of the input data, which can either be SMILES strings or

CDPKit Atom objects (default: "smiles").

- `output`: A list indicating the types of descriptors to generate (default: ["fingerprint", "physicochemical", "topological"]).

This design allows users to customize the descriptor generation process by selecting specific subsets of descriptors or adjusting the fingerprint radius, thereby adapting it to their specific needs.

The `FAME3RScoreEstimator` computes *FAME scores*, which quantify the reliability of predictions based on the average Tanimoto similarity to the k-nearest neighbors in the training data. It is initialized with:

- `n_neighbors`: Specifies the number of nearest neighbors to consider when calculating the *FAME score* (default: 3).

The estimator follows scikit-learn's fit/predict API convention. During the fit step, the estimator stores the training data's feature vectors as a reference. During the predict step, it calculates the Tanimoto similarity between each test sample and all training samples, averaging the top k similarities to produce the *FAME score* for each test sample. *FAME scores* range from 0 to 1, with higher scores indicating greater reliability.

S.6.2 Command Line Interface

The FAME3R Command Line Interface (CLI) offers commands for hyperparameter optimization, model training, threshold tuning, SOM prediction, performance evaluation, and descriptor computation. All commands accept input data in the form of Structure-Data (SD) or Simplified Molecular Input Line Entry System (SMILES) files.

For model training and performance evaluation, experimentally confirmed SOMs must be annotated in the input file under the `soms` property as a list of atom indices. For example, if atoms 1 and 6 are SOMs, the property should be specified as `[1, 6]`. Additional properties in the input file are supported without restrictions on naming or quantity.

The available commands include:

1. `predict`: predict SOMs, and optionally *FAME scores* and Shannon entropy for unlabeled molecules.
2. `train`: build predictive and applicability-scoring models from labeled molecules.
3. `hyperparameters`: optimize model parameters using cross-validation.
4. `threshold`: find the binary decision threshold of a predictive model for optimal performance (post-hoc tuning).
5. `metrics`: compute evaluation metrics, including Area Under the Receiver Operating Characteristic Curve (ROC-AUC), Area Under the Precision Recall Curve (PR-AUC), precision, recall, F1 score, Matthew's Correlation Coefficient (MCC), and Top-2 Correctness Rate (TOP-2).
6. `descriptors`: generate *FAME descriptors* for external use.

A diagram illustrating the interplay between commands is shown in Figure S4. Full command syntax, detailed argument descriptions, and examples are provided in the online documentation.[5]

S.6.3 Graphical User Interface

FAME3R is integrated into the NERDD web platform[6,7](`https://nerdd.univie.ac.at/fame3r`). Users can upload molecular structures, configure prediction settings, and view results directly through the web interface. The Graphical User Interface (GUI) is particularly suited for researchers who are new to computational tools or who wish to quickly test FAME3R's capabilities without setting up a local environment.

S.7 Data

Data for this study were obtained from the October 13, 2023 release[8] of the MetaQSAR database,[9] an expert-curated repository of metabolic information on small organic molecules, primarily xenobiotics, related to phase 1 and phase 2 metabolism observed either *in vivo* or *in vitro*. The data processing pipeline used to convert the raw MetaQSAR data, comprising 2825 parent compounds annotated with 6320 expert-curated SOMs, into the data sets employed for training and validating FAME3R is detailed below.

First, compounds were excluded from the processed data set if they met any of the following criteria. The number of excluded compounds is indicated in parentheses. Note that these steps were applied sequentially, in this exact order.

1. Could not be parsed with RDKit or lacked a valid International Chemical Identifier (InChI) representation (3).
2. Computed InChI did not map to a unique compound identifier (6).
3. Contained elements outside the set H, B, C, N, O, F, Si, P, S, Cl, Br, I (20).
4. Had fewer than 5 heavy atoms (31) or a molecular weight exceeding 1000 Da (13).

Next, the following steps were applied to increase the quality of SOM annotations:

1. **Treatment of uncertain SOMs**: While the original FAME3 model treated uncertain SOMs as equivalent to certain ones, FAME3R excluded them to reduce noise in the data set. This decision resulted in the removal of 397 SOMs and thus a smaller average number of SOMs per compound (2.32 vs. 2.91).

2. **Stereoisomers**: Similar to FAME3, FAME3R does not use stereochemical information, making stereoisomers indistinguishable to the model. This issue was overlooked by the developers of FAME3, resulting in potential overlaps between their splits. We improved the data processing pipeline by merging entries with identical InChI representations after removing stereochemical layers. If duplicates had identical SOMs, one instance was retained; if conflicting SOMs were present, all duplicates were excluded. In total, 502 compounds had at least one duplicate. Out of these, 90 had to be excluded due to conflicting SOM annotations. Overall, the de-duplication strategy led to the removal of 318 compounds.

3. **Refinement of annotations**: Raw SOM labels were carefully inspected and corrected where necessary. Annotations were extended to include topologically equivalent atoms within the same molecule. Specifically, if a non-SOM atom had a topologically identical counterpart labeled as a SOM, it was reclassified as a SOM (666 corrections).

Finally, compounds were standardized and salts or fragments were removed using the ChEMBL Structure Pipeline (standardize_mol() and get_parent_mol()).[10] Additionally, compounds were sanitized using the Chem.SanitizeMol() method from RDKit.[11] The processed data set contains 2,393 compounds. On average, compounds have 2.32 SOMs, with a ratio of SOM to non-SOM heavy atoms of approximately 1:10. The data set does not include non-metabolized compounds due to the scarcity of data on metabolically stable molecules.

To maximize comparability with the FAME3 validation study, the data was divided into training and test sets while preserving the original assignment of compounds wherever applicable. Any new compounds were added exclusively to the training set to maintain consistency between the test sets.

Statistics on the composition of the processed data set used to evaluate FAME3R are presented in Table S3, alongside a comparison with the data used for evaluating the original FAME3 model.

The MetaQSAR database provides detailed information on the reaction type for each biotransformation, categorized into three main reaction classes, which are further divided into 21 subclasses. Table S4 lists these reaction classes and subclasses along with their relative frequencies. These reaction types were used to partition the processed data into four subsets, which were used to train the four versions of the FAME3R models presented in this study. Table S5 summarizes the composition of these subsets: the full dataset, the Phase 1-only subset (redox and hydrolysis reactions), and the Phase 2-only subset (conjugation reactions). For each subset, the table provides key statistics, including the number of substrates, total number of heavy atoms, number of experimentally observed SOMs, average number of SOMs per molecule, and the fraction of SOMs relative to all heavy atoms.

Table S1: SYBYL Atom Types and their CDPKit Designations.

SYBYL Code	Description	CDPKit ID
C.3	sp^3 carbon	1
C.2	sp^2 carbon	2
C.1	sp carbon	3
C.ar	aromatic carbon	4
N.3	sp^3 nitrogen	6
N.2	sp^2 nitrogen	7
N.1	sp nitrogen	8
N.ar	aromatic nitrogen	9
N.am	amide nitrogen	10
N.p13	trigonal nitrogen	11
N.4	quaternary nitrogen	12
O.3	sp^3 oxygen	13
O.2	sp^2 oxygen	14
O.co2	carboxylic oxygen	15
S.3	sp^3 sulfur	18
S.2	sp^2 sulfur	19
S.0	sulfoxide sulfur	20
S.o2	sulfone sulfur	21
P.3	sp^3 phosphorus	22
F	fluorine	23
H	hydrogen	24
Si	silicon	38
Cl	chlorine	47
Br	bromine	48
I	iodine	49
B	boron	54

Table S2: Comparison of the Predictive Performance of FAME3R with FAME fingerprints (FAME3R F) vs. count fingerprints (FAME3R C).

data split	all reactions		phase 1		phase 2	
model	FAME3R F	FAME3R C	FAME3 F	FAME3R C	FAME3 F	FAME3R C
ROC-AUC	0.89 ± 0.01	0.90 ± 0.01	0.88 ± 0.01	0.88 ± 0.01	0.96 ± 0.01	0.97 ± 0.01
PR-AUC	0.55 ± 0.02	0.57 ± 0.02	0.55 ± 0.02	0.56 ± 0.02	0.78 ± 0.03	0.79 ± 0.01
F1 score	0.54 ± 0.01	0.56 ± 0.01	0.53 ± 0.01	0.53 ± 0.02	0.71 ± 0.03	0.72 ± 0.03
MCC	0.49 ± 0.02	0.50 ± 0.02	0.47 ± 0.02	0.48 ± 0.02	0.69 ± 0.03	0.70 ± 0.03
precision	0.51 ± 0.02	0.52 ± 0.02	0.46 ± 0.02	0.46 ± 0.02	0.65 ± 0.03	0.65 ± 0.03
recall	0.57 ± 0.02	0.61 ± 0.02	0.63 ± 0.02	0.65 ± 0.02	0.79 ± 0.03	0.81 ± 0.03
TOP-2	0.78 ± 0.02	0.79 ± 0.02	0.71 ± 0.02	0.73 ± 0.02	0.87 ± 0.02	0.87 ± 0.02

[1]Standard deviations computed from 1,000 bootstrap samples.

Table S3: Comparison of the Composition of the Data Used in this Work vs. in the FAME3 Validation Study.

data split	total		train		test	
model	FAME3[1]	FAME3R	FAME3[1]	FAME3R	FAME3[1]	FAME3R
total no. substrates	2167	2393	1733	2000	434	393
total no. heavy atoms	49045	54340	39131	45480	9914	8860
total no. SOMs	6307	5557	n/a	4613	n/a	944
avg. no. SOMs per molecule	2.91	2.32	n/a	2.31	n/a	2.40
fraction of SOMs among heavy atoms	0.13	0.12	n/a	0.12	n/a	0.13

[1] Values obtained from ref.[2]

Table S4: MetaQSAR Classification of Metabolic Reactions.

**Category**	**ID**	**Description**	**%**
Redox reactions	1	Oxidation of sp^3 hybridized carbon atoms	29.4
	2	Oxidation of sp and sp^2 hybridized carbon atoms	15.5
	3	Redox reactions between alcohols, aldehydes, ketones and carboxylic acids	3.7
	4	Various redox reactions of carbon atoms	1.0
	5	Redox reactions of tertiary amines	2.4
	6	Oxidation of secondary amines, oximes and nitroso etc.; reduction of nitro, nitroso and oximes etc.	3.3
	7	Oxidation to quinones or analogs; reduction of quinones and analogs	2.4
	8	Oxidation and reduction of sulfur atoms	2.9
	9	Redox reactions of other atoms	0.1
Hydrolysis and other reactions	10	Hydrolysis of esters, lactones and inorganic esters	5.3
	11	Hydrolysis of amides, lactams and peptides	2.8
	12	Epoxide hydration	0.3
	13	Other hydrolysis and hydration reactions; nonenzymatic eliminations and rearrangements	2.4
Conjugation reactions	14	Oxygen-glucuronidations and -glycosylations	10.6
	15	Nitrogen- and sulfur-glucuronidations; all other glycosylations	2.8
	16	Sulfonations	2.9
	17	Glutathione and RSH conjugations and their sequels; glutathione-mediated reductions	7.0
	18	Acetylations and acylations	1.6
	19	CoASH-ligation followed by amino acid conjugations or other sequels	1.4
	20	Methylations	1.1
	21	Other conjugations; transaminations	1.0

[1]The union of all "redox reactions" and "hydrolysis and other reactions" constitutes the Phase 1-only data set.

[2]The set of all "conjugation reactions" makes up the Phase 2-only data set.

Table S5: Composition of the Full, CYP-, Phase 1-, and Phase 2-Only Data Sets.

data subset	all	CYP	P1	P2
total no. substrates	2393	1267	1692	1058
total no. heavy atoms	54340	29149	39899	22558
total no. SOMs	5557	3524	4357	1357
avg. no. SOMs per molecule	2.32	2.78	2.58	1.28
fraction of SOMs among heavy atoms	0.12	0.14	0.13	0.08

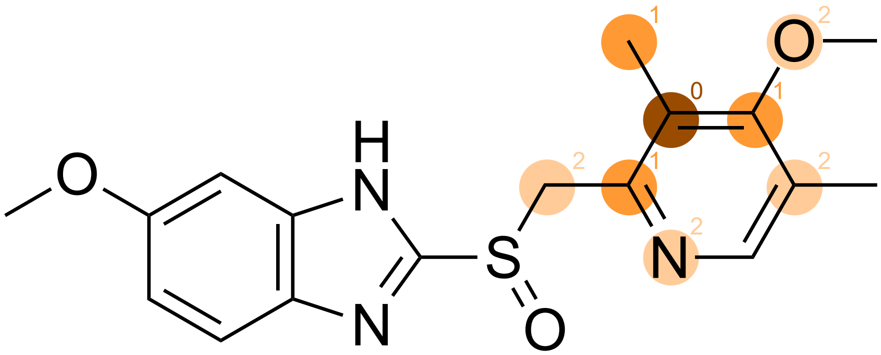

Radius 0

Atom Type	0_C.3	0_C.ar	...	0_N.ar	...	0_O.3
Atom Count	0	1	...	0	...	0
32-Bit Vector	000...000	100...000	...	000...000	...	000...000

Radius 1

Atom Type	1_C.3	1_C.ar	...	1_N.ar	...	1_O.3
Atom Count	1	2	...	0	...	0
32-Bit Vector	100...000	110...000	...	000...000	...	000...000

Radius 2

Atom Type	2_C.3	2_C.ar	...	2_N.ar	...	2_O.3
Atom Count	1	1	...	1	...	1
32-Bit Vector	100...000	100...000	...	100...000	...	100...000

000...000000...000100...000000...000100...000100...000...

(3 radii × 26 atom types × 32 bits = 2496 bits)

Figure S1: **Illustration of the *FAME fingerprint* using omeprazole and a maximum radius of 3.** The atom for which the fingerprint is generated is the aromatic carbon atom (C.ar) highlighted in brown. Therefore, the first bit of C.ar vector at radius zero is activated. In its one-bond neighborhood, the atom has one sp^3-hybridized carbon atom (C.3) and two aromatic carbon atoms, thus activating the first bit of the C.3 vector and the first two bits of the C.ar vector at radius one. No bits are activated for atom types absent within the specified radius. This process is repeated for all radii from zero up to the user-defined maximum radius. Finally, the 32-bit strings for all radii are concatenated to form the *FAME fingerprint*. With a maximum radius set to 3, the final fingerprint contains 4 radii (0 to 3) $\times$ 26 atom types $\times$ 32 bits = 3328 values per atom.

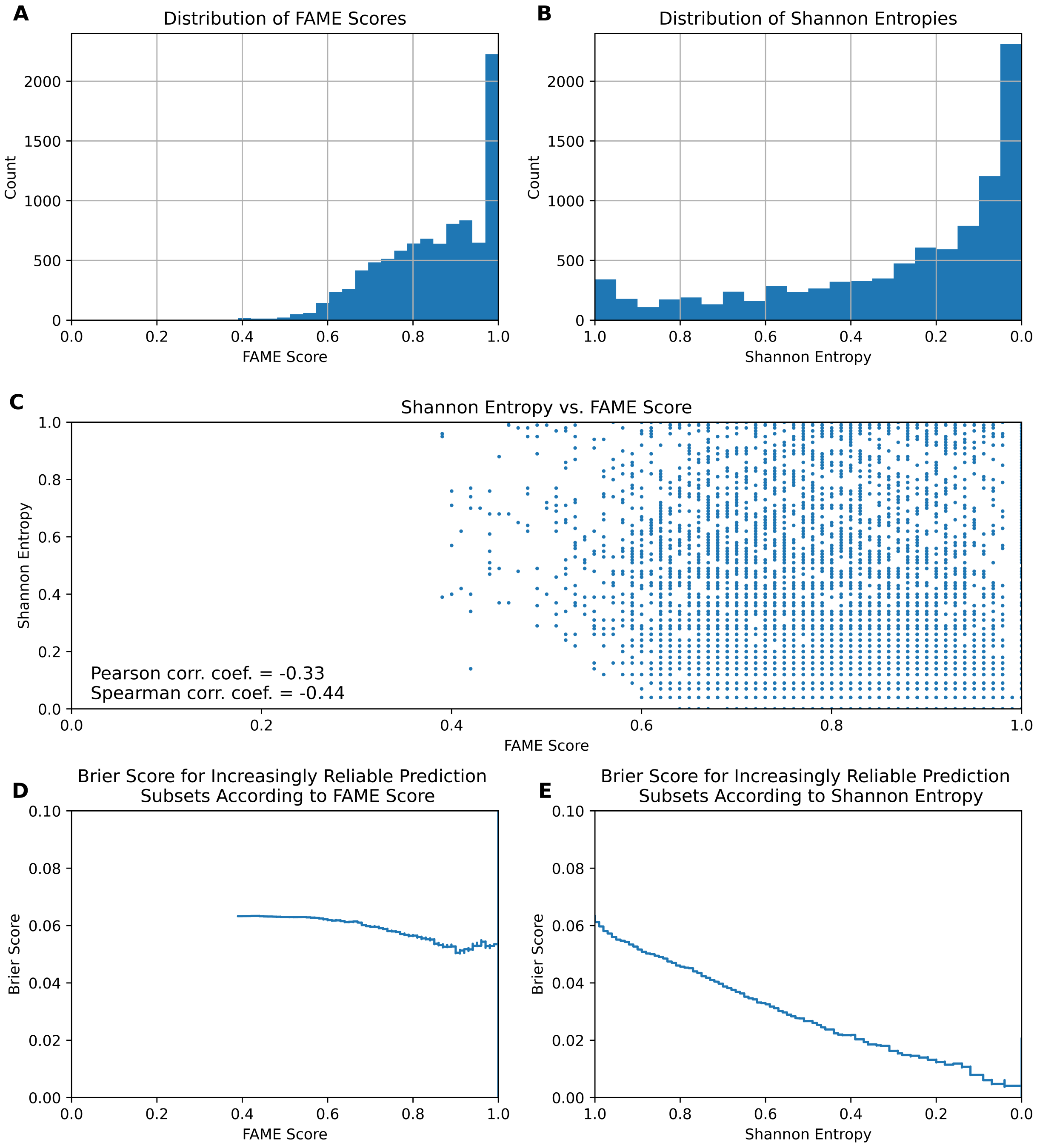

Figure S2: **Comparison of *FAME scores* and Shannon entropy for prediction reliability.** (A) Distribution of *FAME scores*. (B) Distribution of Shannon entropy values. (C) Scatter plot of Shannon entropy versus *FAME score*, showing discrete intervals for *FAME scores* and a lack of strong correlation between the two measures (Pearson correlation: -0.33, Spearman correlation: -0.44). (D, E) Brier score analysis for increasingly reliable prediction subsets ranked by *FAME score* (D) and Shannon entropy (E). The steeper negative slope in (E) indicates that Shannon entropy is a more effective reliability measure, as it incorporates label distribution information from the training set.

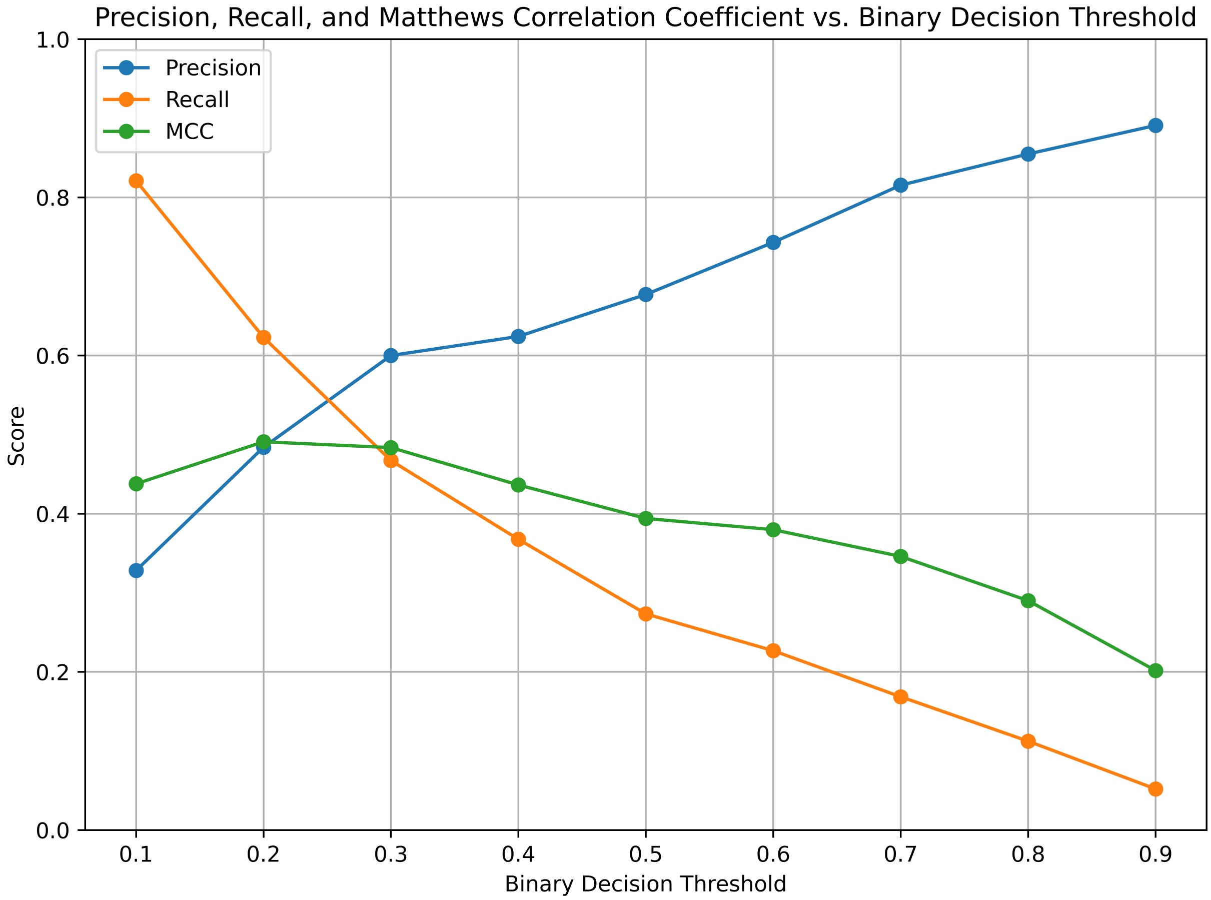

Figure S3: **Precision, recall and MCC vs. binary decision threshold.** The plot demonstrates how adjusting the threshold impacts the trade-off between precision and recall, as well as the overall balance captured by the MCC.

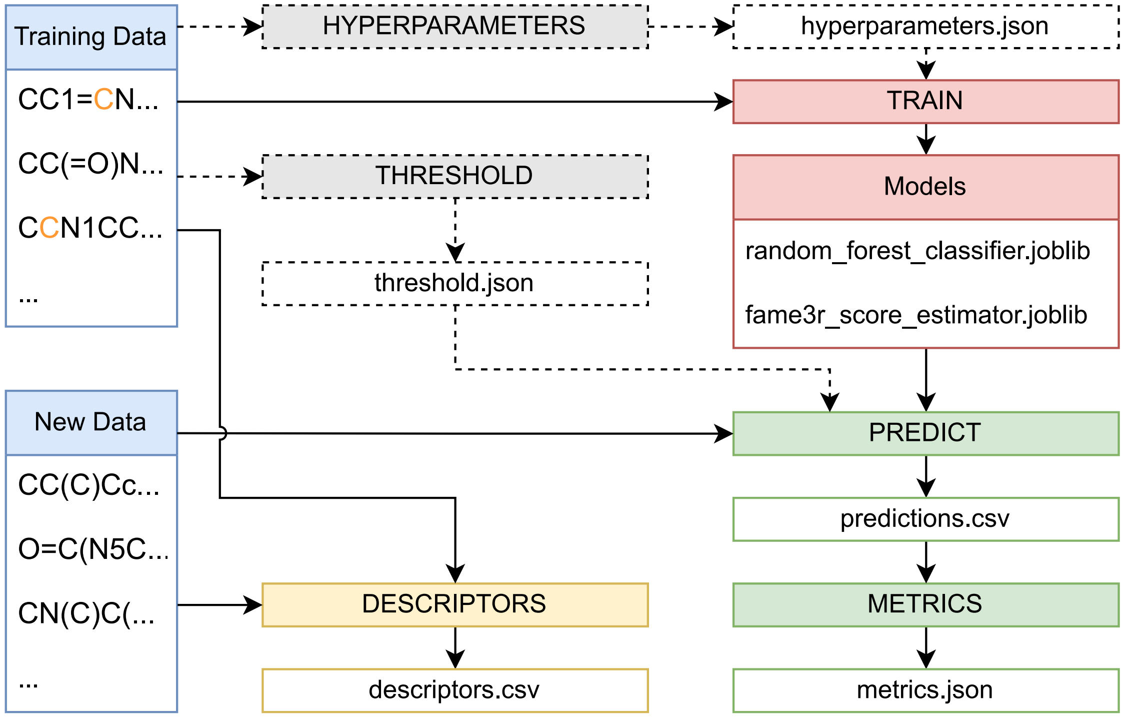

Figure S4: **Workflow diagram of the FAME3R command-line interface tools.** Labeled training data is used to fit two models: a SOM prediction model and a *FAME score* model. Default hyperparameters are provided but can be optimized using the *hyperparameters* command. The trained models are then applied to predict SOMs and *FAME scores* for new data. Binary predictions are derived from probabilistic outputs using a binary decision threshold, which is set to 0.3 by default but can be optimized via the *threshold* command. Notably, the optimization of the SOM predictor's hyperparameters and the binary decision threshold is handled separately, as threshold adjustment is a post-hoc optimization approach distinct from hyperparameter tuning. Dashed lines indicate that these parts of the workflow are optional, since we provide default hyperparameters and a decision threshold. If the new data is labeled, performance metrics can be calculated using the *metrics* command. Additionally, *FAME descriptors* can be generated separately using the *descriptors* command.

References

(1) Seidel, T. Chemical data processing toolkit source code repository. https://github.com/molinfo-vienna/CDPKit, Accessed: 2025-10-25.

(2) Šícho, M.; Stork, C.; Mazzolari, A.; de Bruyn Kops, C.; Pedretti, A.; Testa, B.; Vistoli, G.; Svozil, D.; Kirchmair, J. FAME 3: Predicting the Sites of Metabolism in Synthetic Compounds and Natural Products for Phase 1 and Phase 2 Metabolic Enzymes. *J. Chem. Inf. Model.* **2019**, *59*, 3400–3412.

(3) Clark, M.; Cramer, R. D.; Opdenbosch, N. V. Validation of the general purpose tripos 5.2 force field. *J. of Comput. Chem.* **1989**, *10*, 982–1012.

(4) Pedregosa, F. et al. Scikit-learn: Machine Learning in Python. *Journal of Machine Learning Research* **2011**, *12*, 2825–2830.

(5) Jacob, R. A.; Gaskin, L. FAME 3R: A Fast, Compact, Flexible, and Practical Re-Design of the FAME 3 Model for Predicting Sites-of-Metabolism. https://github.com/molinfo-vienna/FAME3R, Accessed: 2025-10-25.

(6) Hirte, S. NERDD: Next-generation E-Resource for Drug Discovery. https://nerdd.univie.ac.at/, Accessed: 2025-10-25.

(7) Hirte, S.; Scholz, V.-A.; Kirchmair, J. A Scalable Microservices Platform for Deploying Machine Learning Models in Drug Discovery and Beyond. *ChemRxiv* **2025**, doi:10.26434/chemrxiv–2025–thhkd.

(8) Pedretti, A.; Mazzolari, A.; Vistoli, G.; Testa, B. MetaQSAR Database (snapshot from October 13, 2023).

(9) Pedretti, A.; Mazzolari, A.; Vistoli, G.; Testa, B. MetaQSAR: An Integrated Database Engine to Manage and Analyze Metabolic Data. *J. Med. Chem.* **2018**, *61*, 1019–1030.

(10) Bento, A. P.; Hersey, A.; Félix, E.; Landrum, G.; Gaulton, A.; Atkinson, F.; Bellis, L. J.; Veij, M. D.; Leach, A. R. An Open Source Chemical Structure Curation Pipeline Using RDKit. *J. Cheminf.* **2020**, *12*, 51.

(11) RDKit: Open-source cheminformatics. https://www.rdkit.org, Accessed: 2025-10-25.
